# Supplementary material for: Investigating the influence of physiologically relevant hydrostatic pressure on CHO cell batch culture
Source: Sci Rep. 2021 Jan 8;11:162. doi: 10.1038/s41598-020-80576-8 (PMC7794228; doi:10.1038/s41598-020-80576-8)
Supplement: Supplementary file 1 — Supplementary Information 1. [file 41598_2020_80576_MOESM1_ESM.zip › S1/IgG/Feb17 60 90 standard/standard/40ugml volumn.pdf]

===

Acq. Operator : SYSTEM   Seq. Line : 11
Sample Operator : SYSTEM
Acq. Instrument : HPLC-0039   Location : 6
Injection Date : 2/17/2019 6:12:58 PM   Inj : 1
Inj Volume : 100.000 µl
Different Inj Volume from Sample Entry! Actual Inj Volume : 40.000 µl
Sequence File : C:\Users\Public\Documents\ChemStation\1\Data\TK_CHO_IgG 2019-02-17 17-28-56\TK_CHO_IgG.S
Method : C:\Users\Public\Documents\ChemStation\1\Data\TK_CHO_IgG 2019-02-17 17-28-56\CHO_IgG_LC.M (Sequence Method)
Last changed : 2/8/2019 3:17:23 PM by SYSTEM

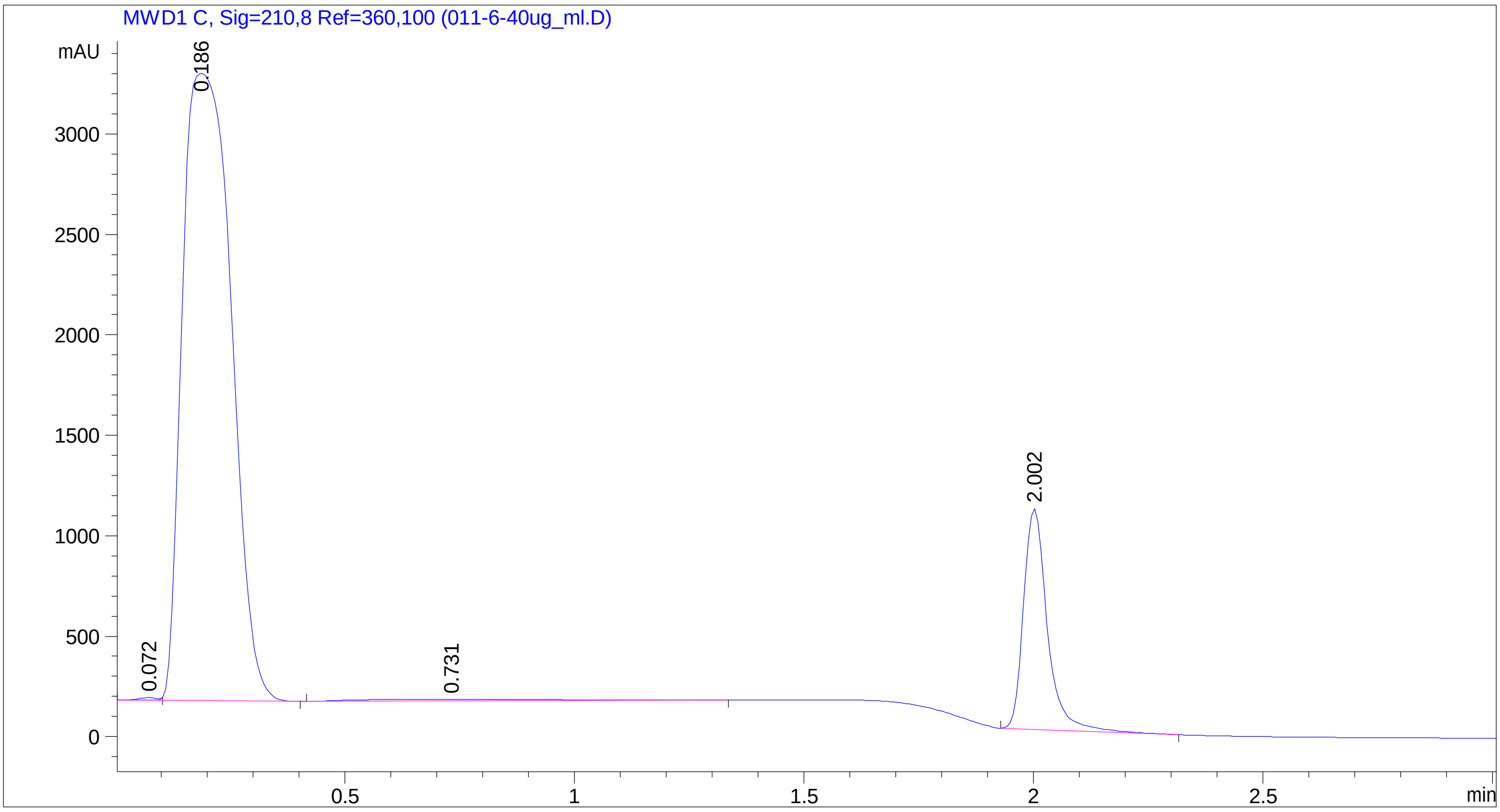

===

Area Percent Report

===

Sorted By : Signal
Multiplier : 1.0000
Dilution : 1.0000
Use Multiplier & Dilution Factor with ISTDs

Signal 1: MWD1 C, Sig=210,8 Ref=360,100

Peak #	RetTime [min]	Type	Width [min]	Area [mAU*s]	Height [mAU]	Area %
1	0.072	BV E	0.0410	33.98977	12.81194	0.1243
2	0.186	VB R	0.1223	2.31041e4	3122.24609	84.4994
3	0.731	BB	0.3884	251.87613	7.80782	0.9212
4	2.002	BBA	0.0539	3952.36792	1100.10706	14.4551

Totals : 2.73423e4 4242.97290

===

*** End of Report ***
